# Symptom Profiles, Health-Related Quality of Life, and Clinical Blood Markers among Korean Community-Dwelling Older Adults Living with Chronic Conditions

**DOI:** 10.3390/ijerph18041745

**Published:** 2021-02-11

**Authors:** Jongmin Park, Nada Lukkahatai, Nancy Perrin, Yoonju Kim, Leorey N. Saligan, Chang Won Won

**Affiliations:** 1 College of Nursing, Pusan National University, Gyeongsangnam-do 50612, Korea; jmpark@pusan.ac.kr; 2 School of Nursing, Johns Hopkins University, Baltimore, MD 21218, USA; nada.lukkahatai@jhu.edu (N.L.); nperrin@jhu.edu (N.P.); 3 Department of Nursing, Graduate School, Kyung Hee University, Seoul 02447, Korea; kimyoonju29@gmail.com; 4 National Institute of Nursing Research, National Institutes of Health, Bethesda, MD 20892, USA; saliganl@mail.nih.gov; 5 Elderly Frailty Research Center, Department of Family Medicine, College of Medicine, Kyung Hee University, Seoul 02447, Korea

**Keywords:** symptom cluster, quality of life, biomarkers, chronic conditions, community-dwelling older adults

## Abstract

Older adults suffer from multiple symptoms, which negatively affects their health-related quality of life. The single-symptom management approach has been less than effective. The data of 2362 Korean community-dwelling older adults aged 70 and above were analyzed in the Korean Frailty and Aging Cohort Study (KFACS) study. A cluster analysis, correlation analysis, and logistic regression were used to analyze the data. We found three symptom clusters: high symptom burden (HSB, *n* = 1032); pain and fatigue group (PAF, *n* = 566); and the sleep deprivation group (SDP, *n* = 764). Participants in the HSB group are more likely to be of old age (OR = 1.1), be female (OR = 2.4), live in a rural area (OR = 1.4), have low physical activity (OR = 0.9), and have multiple chronic conditions (OR = 1.5). The clinical blood markers analysis showed a negative relationship among the physical health, free T4 (*r* = −0.083, *p* < 0.01) and insulin (*r* = −0.084, *p* < 0.01). The sex-specific blood markers analysis showed differences among three clusters. While free testosterone (male: *r* = 0.124, female: *r* = 0.110, *p* < 0.05) and dehydroepiandrosterone (DHEA) (male: *r* = 0.352 and female: *r* = 0.134, *p* < 0.05) were associated with physical health in the HSB group, only free testosterone was associated with mental health (male: *r* = −0.093, and female: *r* = −0.116, *p* < 0.05) in the SDP group. These findings suggest the potential role of the patient’s sex and sex hormones in symptoms of Korean community-dwelling older adults. Understanding the symptom profiles and impact of biopsychosocial factors may enhance precision symptom management.

## 1. Introduction

In 2019, the total aging population in the world was 703 million, which is expected to double by 2050 [1]. As life expectancy increases and the birth rate decreases, the aging population is growing in South Korea. By 2065, the proportion of the elderly will increase three-fold, from 13% in 2015 [2]. Nearly 80% of Korean older adults have at least one chronic condition, including cardiovascular disease, musculoskeletal disease, or diabetes mellitus. With chronic conditions and the aging process, older adults suffer from multiple symptoms such as pain, fatigue, sleep deprivation, and depression [3,4,5]. The intensity of these symptoms negatively affects older adults’ health-related quality of life (HRQoL) [6,7]. Studies suggest that older adults with severe symptom burdens and poor physical function are at risk of hospitalization and nursing home admission [8,9,10,11,12].

The typical approach for symptom management among older adults has been to suggest an intervention based on each individual symptom. This “single symptom” approach results in complicated self-management as older adults and families are required to balance multiple medications, behavioral changes, cognitive coping strategies or physical interventions to manage all symptoms [13]. Many intervention studies (e.g., psychological interventions, physical activity programs, educational programs, massage, acupressure/acupuncture interventions) often find the effect of the interventions on more than one symptom. A symptom cluster is defined as the co-occurrence of two or more symptoms, which may or may not share the same underlying etiology [14]. The identification of symptom clusters in older adults with chronic conditions can be used to develop a simple, personalized, symptom cluster management program [12].

Recent studies suggest the role of inflammatory and endocrine clinical blood markers, including inflammatory cytokines, C-reactive protein, and dehydroepiandrosterone (DHEA) in the development and chronicity of symptoms, particularly with mobility limitation, depression, sleep deprivation, fatigue, and HRQoL [15,16,17,18,19,20,21]. To the best of our knowledge, there is limited evidence on the associations of these clinical blood markers with symptom burden and HRQoL. Investigating the role of these clinical blood markers will increase our understanding of the role of these markers on symptom burden and HRQoL among older adults.

This study aimed to (1) identify symptom clusters based on pain, mobility limitation, fatigue, and sleep deprivation among community-dwelling Korean older adults living with chronic conditions, and (2) investigate the relationships among symptom cluster profiles with HRQoL and clinical blood markers.

## 2. Materials and Methods

### 2.1. Study Population

This is an analysis of baseline data collected from the Korean Frailty and Aging Cohort Study (KFACS), which recruited 3014 community-dwelling older adults aged 70 and older from 10 nationwide hospitals and community health centers in South Korea from 2016 to 2017 [22]. Participants were asked to complete self-reported questionnaires related to socioeconomic status, living environment, health history, health-related behaviors, comorbidities, symptom experiences, and HRQoL. Laboratory tests were conducted to obtain clinical blood marker results. The present study’s inclusion criteria were: (a) individual age 70 years and older, and (b) diagnosed with at least one chronic condition (i.e., cardiovascular disease, cerebrovascular disease, musculoskeletal disease, respiratory disease, gastrointestinal disease, renal disease, endocrine disease, or cancer). Participants who had difficulty communicating, visiting the healthcare center, a history of acute stroke or myocardial infarction during the past six months, or systolic blood pressure over 180 mmHg were excluded from this study.

### 2.2. Measures

#### 2.2.1. Demographic Characteristics

Demographic data, including age, sex, smoking history, alcohol consumption, education, marital status, and living status were obtained from the self-reported questionnaire. Body mass index (BMI) was calculated (kg/m^2^); overweight was defined as a BMI of ≥23 kg/m^2^ [23]. Self-reported physical activities (vigorous, moderate, and light) within the past seven days for at least 10 minutes at a time were measured. 

#### 2.2.2. Symptom Experience

Pain was assessed by a single item: ‘‘How much pain do you have today?" Responses were measured as zero (0) “absence of pain” or one (1) “presence of pain.”

Mobility limitation was measured by the physical functioning (PF) scale, which consisted of 5 items on mobility and self-care [24]. The total scores ranged from 0 to 100, and higher ratings indicated better physical function. The subscale had good internal consistency with Cronbach’s alpha = 0.89 [24] and 0.82. The mobility score of 75.8 [24], was used as a cutoff score for determining participants with “no mobility limitation (0)” and “have mobility limitation (1)”.

Fatigue was measured by a single item: ‘‘Have you ever felt tired in the past month?" The response was measured as zero (0) “absence of fatigue” or one (1) “presence of fatigue.”

Sleep was measured by a single question: “How many minutes of actual sleep do you get at night?” The cutoff sleeping time of 6 hours/day was used to recode this variable into “no sleep deprivation (0)” if a participant slept 6 h or more, and “experienced sleep deprivation (1)” if a participant slept less than 6 h [25].

#### 2.2.3. Health-Related Quality of Life

HRQoL was measured by the 12-Item Short-Form Health Survey (SF-12), which was developed to be a shorter version of SF-36, which is the most commonly used as a generic health status questionnaire [26]. The questionnaire consists of 12 items with eight concepts: physical functioning (two items), role-physical (two items), bodily pain (one item), general health (one item), vitality (one item), social functioning (one item), role-emotional (two items), and mental health (two items). The summary scales are referred to as the physical component summary (PCS-12) and the mental component summary (MCS-12) scale scores. Higher scores indicate better HRQoL. Cronbach’s alpha for PCS-12 and MCS-12 in the general Korean population was 0.83 and 0.79 [27] and in our study was 0.84 and 0.82, respectively.

#### 2.2.4. Clinical Blood Markers

Participants’ peripheral blood samples were taken at about 08:00 a.m. after 8 h of fasting. All blood samples at the ten centers were taken to a commercial laboratory for analysis. The white blood cell (WBC) count was measured using Cellpack DCL diluent (Sysmex, Co., Kobe, Japan) on an XN-9000 hematology analyzer (Sysmex Co., Kobe, Japan). The concentration of high-sensitivity C-reactive protein (hs-CRP) was determined using CRPHS reagent (Roche, Mannheim, Germany) on a Cobas 8000 C702 (Roche, Mannheim, Germany). Thyroid-stimulating hormone (TSH), Free T4, and insulin were measured by Cobas 8000 e602 (Roche, Mannheim, Germany) using each reagent. The radioimmunoassay (RIA) method was used to analyze free testosterone using 1470 Wizard ϒ-counter (PerkinElmer, Turku, Finland) with a free testosterone RIA CT kit (Asbach Medical Products, Obrigheim, Germany). Insulin-like growth factor (IGF-1) and dehydroepiandrosterone (DHEA) were measured by a microplate reader (VERSAmax, Sunnyvale, USA) with an IGF-1 enzyme-linked immunosorbent assay (ELISA) kit (Mediagnost, Reutlingen, Germany) and DHEA ELISA kit (IBL International, Hamburg, Germany).

### 2.3. Ethical Considerations

The KFACS was approved by the institutional review board (IRB) at the Kyung Hee Medical Center, Seoul, Republic of Korea (KHUH 2015-12-103). As a secondary analysis using de-identified data, this study was exempt from IRB review (KHSIRB-20-234).

### 2.4. Statistical Analyses

The G*Power (version 3.1.9.5., University of Dusseldorf, Dusseldorf, Germany) software was used to calculate the sample size for this study. The expected correlation between clinical blood markers and HRQoL was 0.1. With an alpha value of 0.05 and a power of 0.95, we calculated the estimated sample size to be 1077 [28]. All statistical analyses were performed using SPSS version 25.0 (IBM SPSS Statistics, SPSS, Chicago, IL, USA). A *p*-value of ≤0.05 was considered statistically significant. All data were presented as the mean (standard deviation (SD)) and percentage. Hierarchical cluster analysis was used to identify the subgroups of symptom clusters. Hierarchical cluster analysis was performed using Ward’s method on squared Euclidian distances using the four symptoms: pain, mobility limitation, fatigue, and sleep deprivation. The appropriate number of clusters was selected using the agglomeration schedule. The subgroups were compared to demographic characteristics using the chi-square test, one-way analysis of variance (ANOVA) and the Kruskal–Wallis test. We performed logistic regression to identify the associated factors, including socio-demographics and the presence of chronic conditions, on the likelihood of a high symptom burden. Data were expressed as odds ratios (ORs) with 95% confidence intervals (CIs). We performed Spearman’s rank correlation analysis to explore the relationships between the number of symptoms, HRQoL, and biomarkers.

## 3. Results

### 3.1. General Characteristics of Participants

A total of 2362 participants who met the inclusion criteria were included in this analysis. More than 55% of the samples were females with a mean age of 76.1 (3.9) and 8.55 (5.72) of education. The majority of the participants were married (66%), living with others (76%), and residing in an urban area (73%). More than 70% were overweight (BMI ≥ 23.0). Participants reported that they had light physical activity more than 5 days/week on average. More than 80% of the participants were diagnosed with cardiovascular disease. Nearly 40% of the participants had a musculoskeletal disease. The participants who were diagnosed with cerebrovascular, respiratory, gastrointestinal, neurological disease, and cancer were less than 10%. Nearly 60% were living with at least two chronic illnesses (Table 1).

### 3.2. Description of Symptom Clusters

The cluster analysis of the symptoms (pain, mobility limitation, fatigue, and sleep deprivation) showed three symptom cluster profiles: subgroup 1, high symptom burden (HSB) group (*n* = 1032, 43.7%); subgroup 2, pain and fatigue (PAF) group (*n* = 566, 24.0%); and subgroup 3, sleep deprivation (SDP) group (*n* = 764, 32.3%) (Table 2). Among the three subgroups, participants in the PAF and SDP subgroups were younger (*p* < 0.001), reported a higher number of days of physical activity (*p* < 0.001), and had a higher proportion of participants with only one chronic condition than the HSB subgroup (*p* < 0.001) (Table 1).

### 3.3. Predicting Variables with High Symptom Burden

We selected the potential associated factors that were significantly different between groups. The full model containing all predictors was statistically significant, *χ*^2^ (14, *n* = 2362) = 569.05 (*p* < 0.001). The model that explained the Cox and Snell R square was 22%, and Nagelkerke R squared was 29%. We found that the following were significant contributors to the model: age (OR = 1.11), being female (OR = 2.40), BMI (OR = 1.05), non-smoker (OR = 1.51), moderate physical activity (OR = 0.95), light physical activity (OR = 0.89), years of education (OR = 0.93), living in a rural area (OR = 1.36), and living with chronic illnesses for more than 2 years (OR = 1.52) (Table 3). Sex was the strongest predictor of high symptom burden, recording an odds ratio of 2.40. This result indicated that females were over 2.40 times more likely to have a high symptom burden than men, controlling for all other factors in the model.

### 3.4. Correlations of Health-Related Quality of Life and Clinical Blood Markers among Different Symptom Clusters

The results of the present study show the unique associations of clinical blood markers and HRQoL among these three symptom cluster subgroups (Table 4). Although no significant associations among HRQoL and clinical blood markers were shown in the PAF cluster, the insulin, free testosterone, and DHEA levels were significantly associated with either physical health or mental health in the HSB and SDP clusters. In the HSB cluster, physical health was negatively associated with free T4 (*r* = −0.083, *p* < 0.01) and insulin (*r* = −0.084. *p* < 0.01) levels. In the SDP cluster, physical health was negatively associated with WBC (*r* = −0.086, *p* < 0.05) and insulin (*r* = −0.111, *p* < 0.01).

We also found that the relationships among sex-specific blood markers and HRQoL components were different among the three cluster groups. While there were no significant correlations of the clinical blood makers and HRQoL in the PAF group, in the HSB group, low free testosterone (male: *r* = 0.124, female: *r* =0.110, *p* < 0.05) and low DHEA (male: *r* = 0.352 and female: *r* =0.134, *p* < 0.05) were associated with low physical health. In the SDP group, free testosterone was significantly associated only with the mental health component of HRQoL for both male (*r* = −0.093, *p* < 0.05) and female (*r* = −0.116, *p* < 0.05) participants (Table 5).

## 4. Discussion

Among the Korean community-dwelling older adults, we found three clusters with different symptom compositions, including pain, mobility limitation, fatigue, and sleep deprivation. Pain, fatigue, mobility problems, and sleep deprivation are common in most chronic conditions, such as chronic obstructive pulmonary disease, chronic kidney disease, and lung cancer [7,29,30,31]. Our study provides a unique perspective on the subgroups of these symptoms including the high symptoms burden subgroup (HSB: 44% of our participants), the pain and fatigue subgroup (PAF: 24%), and the sleep deprivation subgroup (SDP: 32%).

Similar to other studies which enrolled participants with various chronic conditions such as end-stage renal disease, atrial fibrillation, and lung cancer [32,33,34,35,36], we found that age, sex, and having multiple chronic conditions were associated with high symptom burden and poor HRQoL. We also found that more than 24% of our participants mainly suffered from pain and fatigue (PAF subgroup) and that 32% mainly suffered from sleep deprivation (SDP subgroup). The participants in these two subgroups (PAF and SDP) were younger, reported a higher number of days engaged in physical activity, and were more likely having only one chronic condition than the HSB subgroup. Consistently with our findings, a national survey of community-dwelling older adults in the United States showed that older adults who suffered from one or two symptoms were younger and had a small number of chronic conditions than the over three symptoms group [37]. This finding might introduce symptoms profiling as an alternative way to tailor interventions such as exercise programs for people who may have multiple chronic conditions. Age, active lifestyle and the number of underlying chronic conditions were significant predictors of the success of many rehabilitation programs [38,39]; however, tailored programs based on age, lifestyle and the number of chronic conditions can be challenging. Thus, we suggest that a tailored intervention program is needed to efficiently manage symptoms for older adults living with chronic conditions.

Studies have suggested sex differences in symptoms experience [40,41,42]. We found that sex was the strongest predictor of high symptom burden. Females were two-fold more likely to experience a high symptom burden than males. Other studies among older adults in Japan [43], Singapore [44], and South Korea [45,46,47] reported that females have higher levels of symptoms (e.g., pain, fatigue, depression and sleep disturbance) than their male counterparts. The mechanism of higher symptoms burden in females is unclear. Studies have suggested that the role of gonadal sex steroids (e.g., estrogen, progesterone and testosterone) levels may influence their symptoms experiences [48,49,50]. Our study found that high sex hormones (free testosterone and DHEA) were significantly associated with the high HRQoL physical component in both the HSB and SDP subgroups but not in the PAF subgroup. These associations were significant even after we divided the subgroups based on sex. Our finding provides additional evidence to support the role of testosterone, a male sex hormone, in maintaining physical function and the perceived quality of life [50,51].

The DHEA hormone is an essential precursor for the production of female sex hormones—estrogen and androgen [52]—and has long been known for its ability to improve depressive symptoms [53]. Another study found the significant correlation between low DHEA and poor physical function [19]. Our study found that high DHEA levels were significantly associated with the high HRQoL—physical component, though only in the HSB subgroup for both male and female participants. Only the SDP subgroup showed a significant correlation between high DHEA levels and low HRQoL—mental component, but only among male participants. No significant correlation between DHEA and HRQoL was found in the PAF subgroup. These results suggest the unique role of DHEA in the symptoms experienced between the sexes, which may explain why people respond to DHEA supplementation differently. DHEA supplementation increased the plasma level of testosterone in both males and females, but the prominent increase was shown in female, healthier and younger participants [54,55].

We acknowledge the number of limitations of our study, which include: (a) the cross-sectional nature of the study; (b) the symptoms (pain, fatigue, and sleep deprivation) being measured by single-item and dichotomous questions; (c) the homogenous population that may only be generalized to the Korean community-dwelling older adults; (d) patients with acute stroke or myocardial infarction during the past six months, or systolic blood pressure over 180 mmHg were excluded due to the parent study exclusion criteria; and (e) only inflammatory and endocrine biomarkers were included in the analysis. However, our study provides an initial glimpse of various symptom cluster profiles that have an impact on HRQoL, as influenced by the clinical blood markers collected from a large nationwide sample size (*n* = 2362). Our study provides a unique perspective on predictive factors for high symptom burden and the roles of sex-specific hormones among older adults. Further studies are required to further explore the relationships revealed in this study.

## 5. Conclusions

Korean community-dwelling older adults living with chronic conditions suffer from various symptoms that reduce their HRQoL: pain, limited mobility, fatigue, and sleep deprivation. Our findings suggested the potential role of sex and sex hormones such as DHEA in the symptoms of Korean community-dwelling older adults. In terms of symptom management, understanding unique symptom cluster profiles and their predictive markers is worthwhile to develop optimal and precise interventions.

## Figures and Tables

**Table 1:** Demographics and general characteristics of the symptom cluster subgroups.

Variables	Categories	Total (*n* = 2362)	HSB (*n* = 1032)	PAF (*n* = 566)	SDP (*n* = 764)	*p*
		M(SD)/*n* (%)	M(SD)/*n* (%)	M(SD)/*n* (%)	M(SD)/*n* (%)	
Age (years) ^†^		76.1 (3.9)	76.9 (3.9)	75.6 (3.8)	75.3 (3.67)	<0.001
Sex						<0.001
	Males	1053 (44.6)	261 (25.3)	326 (57.6)	466 (61.0)	
	Females	1309 (55.4)	771 (74.7)	240 (42.4)	298 (39.0)	
Body mass index						0.004
	Underweight	36 (1.5)	20 (1.9)	10 (1.8)	6 (0.8)	
	Normal	654 (27.7)	250 (24.2)	178 (31.4)	226 (29.6)	
	Overweight	1672 (70.8)	762 (73.8)	378 (66.8)	532 (70.8)	
Smoking						<0.001
	Ever	851 (36.1)	214 (20.8)	259 (45.8)	378 (49.6)	
	Never	1507 (63.9)	816 (79.2)	307 (54.2)	384 (50.4)	
Alcohol consumption						<0.001
	Ever	1646 (69.8)	635 (61.7)	419 (74.2)	592 (77.6)	
	Never	712 (30.2)	395 (38.3)	146 (23.9)	171 (22.4)	
Physical activity ^†^ (days per week)	Vigorous	0.31 (1.13)	0.17 (0.88)	0.37 (1.25)	0.45 (1.30)	<0.001
	Moderate	2.51 (2.61)	2.10 (2.50)	2.74 (2.60)	2.89 (2.69)	<0.001
	Light	5.36 (2.29)	4.98 (2.51)	5.51 (2.15)	5.77 (1.99)	<0.001
Education (years) ^†^		8.55 (5.72)	6.70 (6.36)	9.59 (4.68)	10.27 (4.73)	<0.001
Marital status						<0.001
	Married	1547 (65.6)	536 (52.0)	426 (75.3)	585 (76.7)	
	Divorced and widowed	813 (34.4)	495 (48.0)	140 (24.7)	178 (23.3)	
Living status						<0.001
	With others	1803 (76.3)	691 (67.0)	464 (82.0)	648 (84.8)	
	Alone	559 (23.7)	341 (33.0)	102 (18.0)	116 (15.2)	
Living area						<0.001
	Urban	1724 (73.4)	711 (69.2)	400 (70.9)	613 (80.8)	
	Rural	626 (26.6)	316 (30.8)	164 (29.1)	146 (19.2)	
Home ownership						<0.001
	Owner occupied	1893 (80.2)	771 (74.9)	478 (84.5)	644 (84.3)	
	Rented	466 (19.7)	258 (25.1)	88 (15.5)	120 (15.7)	
Cardiovascular disease						
	Yes	1934 (81.9)	842 (81.6)	465 (82.2)	627 (82.1)	0.948
	No	428 (18.1)	190 (18.4)	101 (17.8)	137 (17.9)	
Cerebrovascular disease						0.864
	Yes	135 (5.7)	56 (5.4)	34 (6.0)	45 (5.9)	
	No	2227 (94.3)	976 (94.6)	532 (94.0)	719 (94.1)	
Musculoskeletal disease						<0.001
	Yes	992 (42.0)	592 (57.4)	188 (33.2)	212 (27.7)	
	No	1370 (58.0)	440 (42.6)	378 (66.8)	552 (72.3)	
Respiratory disease						0.417
	Yes	179 (7.6)	86 (8.3)	42 (7.4)	51 (6.7)	
	No	2183 (92.4)	946 (91.7)	524 (92.6)	713 (93.3)	
Gastrointestinal disease						<0.001
	Yes	190 (8.0)	107 (10.4)	49 (8.7)	34 (4.5)	
	No	2172 (92.0)	925 (89.6)	517 (91.3)	730 (95.5)	
Neurological disease						0.407
	Yes	21 (0.9)	11 (1.1)	5 (0.9)	5 (0.7)	
	No	2341 (99.1)	1021 (98.9)	561 (99.1)	759 (99.3)	
Renal disease						0.070
	Yes	44 (1.9)	15 (1.5)	17 (3.0)	12 (1.6)	
	No	2318 (98.1)	1017 (98.5)	549 (97.0)	752 (98.4)	
Endocrine disease						0.444
	Yes	712 (30.1)	297 (28.8)	177 (31.3)	238 (31.2)	
	No	1650 (69.9)	735 (71.2)	389 (68.7)	526 (68.8)	
Cancer						0.257
	Yes	91 (3.9)	33 (3.2)	22 (3.9)	36 (4.7)	
	No	2271 (96.1)	999 (96.8)	544 (96.1)	728 (95.3)	
Number of chronic illnesses						<0.001
	1	979 (41.4)	336 (32.6)	256 (45.2)	387 (50.7)	
	≥2	1383 (58.6)	696 (67.4)	310 (54.8)	377 (49.3)	

Note: HSB, high symptom burden; PAF, pain and fatigue; SDP, sleep deprivation; data were presented as the mean (standard deviation (SD)) or a percentage. The *p*-values were based on the chi-square test, one-way analysis of variance (ANOVA), and the Kruskal–Wallis test ^†^.

**Table 2:** Classification of symptoms in the symptom cluster subgroups.

Variables	Total	HSB	PAF	SDP	*p*
Total number of participants	2362	1032 (43.7%)	566 (24.0%)	764 (32.3%)	
Pain	1191	793 (66.6%)	398 (33.4%)	0 (0.0%)	<0.001
Mobility limitation	773	773 (100%)	0 (0.0%)	0 (0.0%)	<0.001
Fatigue	959	649 (67.7%)	310 (32.3%)	0 (0.0%)	<0.001
Sleep deprivation	834	583 (69.9%)	0 (0.0%)	251 (30.1%)	<0.001

Note: HSB, high symptom burden; PAF, pain and fatigue; SDP, sleep deprivation.

**Table 3:** Logistic regression predicting the likelihood of high symptom burden.

Variables	OR	95% CI	*p*
Age	1.113	1.085–1.142	<0.001
Female	2.398	1.715–3.354	<0.001
Body mass index	1.049	1.016–1.083	0.003
Non smoker	1.508	1.094–2.079	0.012
Non alcohol drinker	1.035	0.835–1.284	0.751
Days of vigorous physical activity	0.911	0.829–1.001	0.054
Days of moderate physical activity	0.952	0.917–0.988	0.009
Days of light physical activity	0.886	0.850–0.924	<0.001
Rural	1.356	1.083–1.698	0.008
Yeas of education	0.932	0.911–0.952	<0.001
Divorced and widowed	1.024	0.753–1.392	0.882
Alone	1.170	0.848–1.614	0.339
Rented	1.210	0.947–1.547	0.128
Number of chronic illnesses ≥ 2	1.517	1.251–1.839	<0.001

Note: OR, odds ratio; CI, confidence interval.

**Table 4:** Associations of symptom clusters, health-related quality of life, and clinical blood markers.

Subgroups	Variables	Mean (SD)	Spearman’s Rho							
			PCS-12	MCS-12	WBC	hs-CRP	TSH	Free T4	Insulin	IGF-1
High symptoms burden	1. PCS-12	35.33 (9.89)	1	0.093 **	−0.049	−0.038	0.043	−0.083 **	−0.084 **	0.052
	2. MCS-12	49.18 (11.63)		1	0.025	0.055	0.014	0.032	0.011	−0.063
	3. WBC (1000/µL)	5.98 (1.77)			1	0.203 **	−0.068 *	0.113 **	0.214 **	0.083
	4. hs-CRP (mg/L)	1.39 (1.92)				1	−0.02	−0.01	0.089 **	0.02
	5. TSH (uIU/mL)	2.80 (5.31)					1	−0.300 **	−0.012	−0.069
	6. Free T4 (ng/dL)	1.23 (0.19)						1	−0.061	−0.008
	7. Insulin (uU/mL)	8.83 (11.40)							1	0.037
	8. IGF-1 (ng/mL)	108.37 (58.52)								1
Pain and fatigue	1. PCS-12	44.99 (8.53)	1	−0.006	−0.033	−0.011	0.029	−0.045	−0.036	−0.005
	2. MCS-12	52.98 (9.09)		1	0.047	0.003	−0.057	0.04	0.027	0.075
	3. WBC (1000/µL)	6.05 (1.53)			1	0.191 **	−0.079	0.159 **	0.202 **	0.014
	4. hs-CRP (mg/L)	1.39 (2.23)				1	0.087 *	−0.068	0.004	−0.057
	5. TSH (uIU/mL)	2.71 (4.43)					1	−0.253 **	−0.05	−0.14
	6. Free T4 (ng/dL)	1.24 (0.21)						1	−0.125 **	0.135
	7. Insulin (uU/mL)	8.08 (9.34)							1	0.021
	8. IGF-1 (ng/mL)	123.68 (68.35)								1
Sleep deprivation	1. PCS-12	49.73 (6.53)	1	−0.185 **	−0.016	−0.086 *	0.025	0.005	−0.111 **	0.077
	2. MCS-12	56.09 (7.13)		1	−0.022	0.048	0.025	−0.019	0.037	−0.114
	3. WBC (1000/µL)	5.83 (1.90)			1	0.175 **	0.012	0.079 *	0.197 **	0.049
	4. hs-CRP (mg/L)	1.35 (2.07)				1	0.036	−0.011	0.147 **	−0.039
	5. TSH (uIU/mL)	2.68 (2.54)					1	−0.334 **	−0.039	−0.065
	6. Free T4 (ng/dL)	1.23 (0.20)						1	−0.085 *	0.044
	7. Insulin (uU/mL)	7.67 (9.02)							1	−0.029
	8. IGF-1 (ng/mL)	123.13 (66.12)								1

Note: SD, standard deviation; PCS-12, physical component summary of the 12-Item Short Form Health Survey; MCS-12, mental component summary of the 12-Item Short Form Health Survey; WBC, white blood cell; hs-CRP, high sensitivity C reactive protein; TSH, thyroid stimulating hormone; IGF-1, insulin-like growth factor I; * *p* < 0.05; ** *p* < 0.01.

**Table 5:** Associations of symptoms clusters, health-related quality of life, and sex-specific clinical blood markers.

Subgroups	Sex	Variables	Mean (SD)	Spearman’s Rho			
				PCS-12	MCS-12	Free Testosterone	DHEA
High symptoms burden	Male	1. PCS-12	37.77 (10.62)	1	0.008	0.124 *	0.352 **
		2. MCS-12	50.59 (10.90)		1	0.077	−0.097
		3. Free testosterone (pg/mL)	8.85 (3.61)			1	0.510 **
		4. DHEA (ng/mL)	0.86 (0.57)				1
	Female	1. PCS-12	34.51 (9.50)	1	0.107 **	0.110 **	0.134 *
		2. MCS-12	48.70 (11.83)		1	0.062	−0.037
		3. Free testosterone (pg/mL)	0.83 (0.71)			1	0.643 **
		4. DHEA (ng/mL)	0.75 (0.58)				1
Pain and fatigue	Male	1. PCS-12	45.09 (8.70)	1	0.018	0.031	0.007
		2. MCS-12	52.82 (9.40)		1	−0.075	−0.077
		3. Free testosterone (pg/mL)	9.06 (3.29)			1	0.384 **
		4. DHEA (ng/mL)	0.99 (1.03)				1
	Female	1. PCS-12	44.86 (8.32)	1	−0.038	0.004	0.155
		2. MCS-12	53.21 (8.67)		1	0.02	0.01
		3. Free testosterone (pg/mL)	0.96 (1.00)			1	0.659 **
		4. DHEA (ng/mL)	0.80 (0.64)				1
Sleep deprivation	Male	1. PCS-12	50.50 (6.11)	1	−0.182 **	0.063	0.066
		2. MCS-12	56.50 (6.26)		1	−0.093 *	−0.161 *
		3. Free testosterone (pg/mL)	9.33 (3.29)			1	0.381 **
		4. DHEA (ng/mL)	0.98 (0.67)				1
	Female	1. PCS-12	48.52 (6.98)	1	−0.194 **	0.102	−0.021
		2. MCS-12	55.45 (8.29)		1	−0.116 *	−0.155
		3. Free testosterone (pg/mL)	0.94 (0.89)			1	0.580 **
		4. DHEA (ng/mL)	1.00 (1.16)				1

Note: SD, standard deviation; PCS-12, physical component summary of the 12-Item Short Form Health Survey; MCS-12, mental component summary of the 12-Item Short Form Health Survey; DHEA, dehydroepiandrosterone; * *p* < 0.05; ** *p* < 0.01.

## Data Availability

Data sharing is not applicable to this article.
